# Tumour or Tooth? A Case of Missed Tooth Aspiration

**DOI:** 10.7759/cureus.4267

**Published:** 2019-03-19

**Authors:** Jake Melhuish, James Fearnley, Nazrudeen Ali, Laetitia Jervis

**Affiliations:** 1 Orthopaedics, University Hospitals of Leicester, Leicester, GBR; 2 Anesthesiology, Northampton General Hospital, Northampton, GBR; 3 Paediatrics, University of East Anglia, Norwich, GBR

**Keywords:** tooth, chest radiograph, aspiration, critical care, extubation, bronchoscopy

## Abstract

Aspiration of teeth is a rare, potentially fatal complication of tracheal intubation. Early diagnosis and treatment are key to preventing further complications. Interpretation of the physical examination and radiographic evidence together with a high degree of suspicion are necessary to achieve early diagnosis of foreign body aspiration. We examine one such case of a misdiagnosed tooth in lung event.

## Introduction

Tracheal intubation carries many risks; one rare and potentially fatal complication is the aspiration of dislodged teeth. Early diagnosis and intervention are required to prevent the complete obstruction of the affected airway. A high degree of suspicion and interpretation of the physical examination and radiographic evidence are required to achieve early diagnosis of foreign body aspiration. It is noted that foreign body aspiration can be tolerated in semi-conscious or unconscious patients for a long time and that it is sometimes difficult to interpret the radiology screening performed. We examine one such case of a misdiagnosed tooth in lung event secondary to tracheal intubation in the intensive care setting.

## Case presentation

A 70-year-old man was admitted for an elective right hemicolectomy for a large bowel tumour. He was previously fit and well and lived independently. He smoked 12 cigarettes a day and drank 30 units of alcohol per week on average.

His operation was uneventful. He underwent a laparoscopic right hemicolectomy for stenosing cancer of the hepatic flexure. The anaesthetic record states a grade one laryngoscopy with dentures and few native teeth. He was extubated post-surgery and was admitted to the high dependency unit for 24 hours. He was then discharged to the surgical level 1 facility.

One day after discharge to the level 1 facility, the patient underwent further emergency laparotomy for an anastomotic leak. On the anaesthetic chart, it was noted that a left upper tooth was very loose. This was removed on induction and kept. Intubation proceeded uneventfully.

Postoperatively, he was kept sedated and intubated due to the high risk of further deterioration because of bowel contents soiling during the operation. The plain film chest X-ray taken showed no abnormality. As he was stable, he was extubated that afternoon.

**Figure 1:**
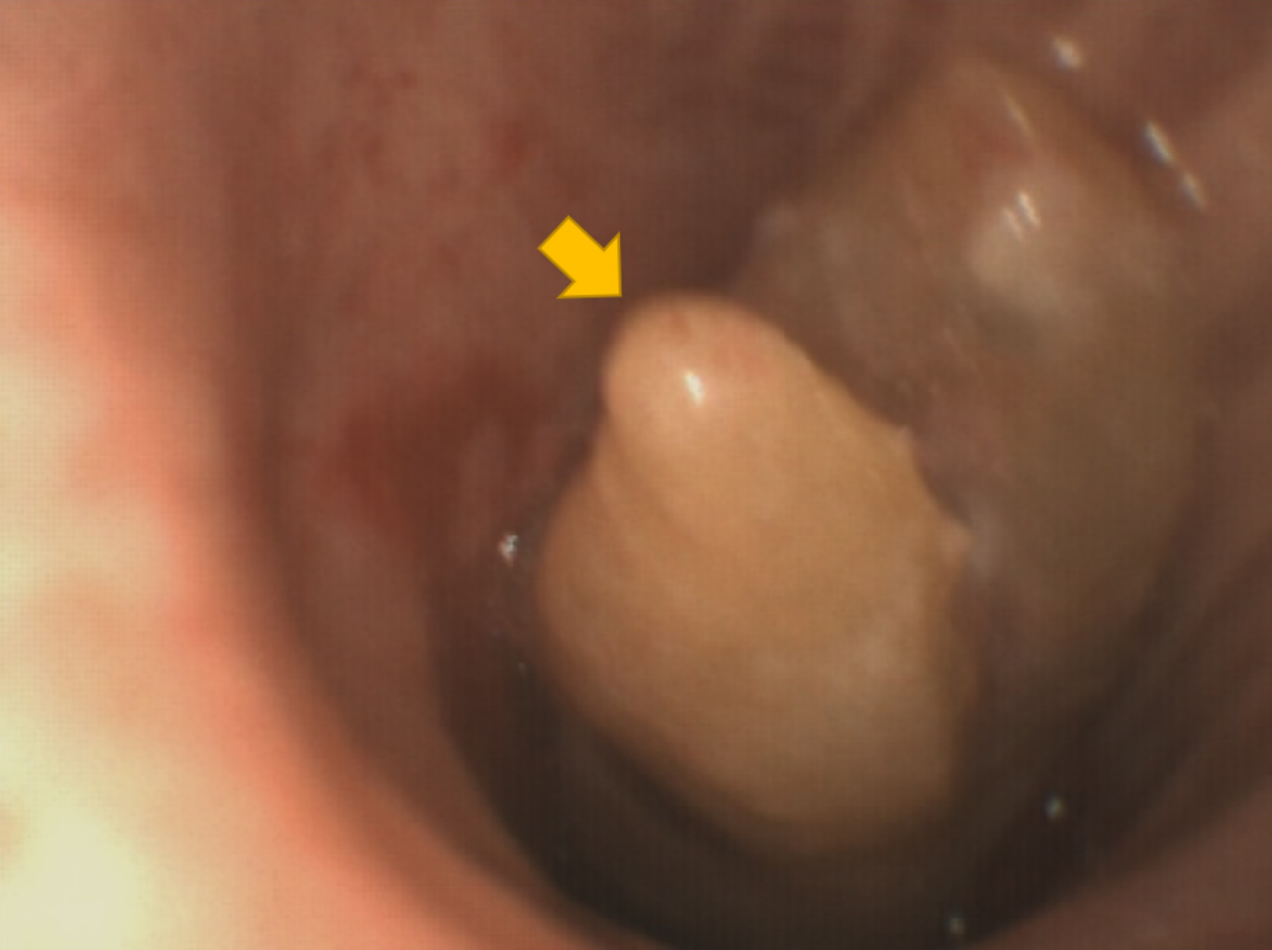
Tooth in bronchus as seen on bronchoscopy.

The following morning, he desaturated and was reintubated. Flexible bronchoscopy was performed due to capillary oxygen saturation (SpO2) 70% with the fraction of inspired oxygen being (FiO2) 100%. There was an unexpected finding of a large tissue mass located 5 cm into the left main bronchus; it was difficult to pass the bronchoscope past this lesion. This lesion is shown in Figure 1. After suctioning the copious secretions, his oxygen saturation recovered to 100% on FiO2 50%. The ‘tissue mass’ would subsequently prove to be a tooth but, at the time, was thought to be a neoplasm.

**Figure 2:**
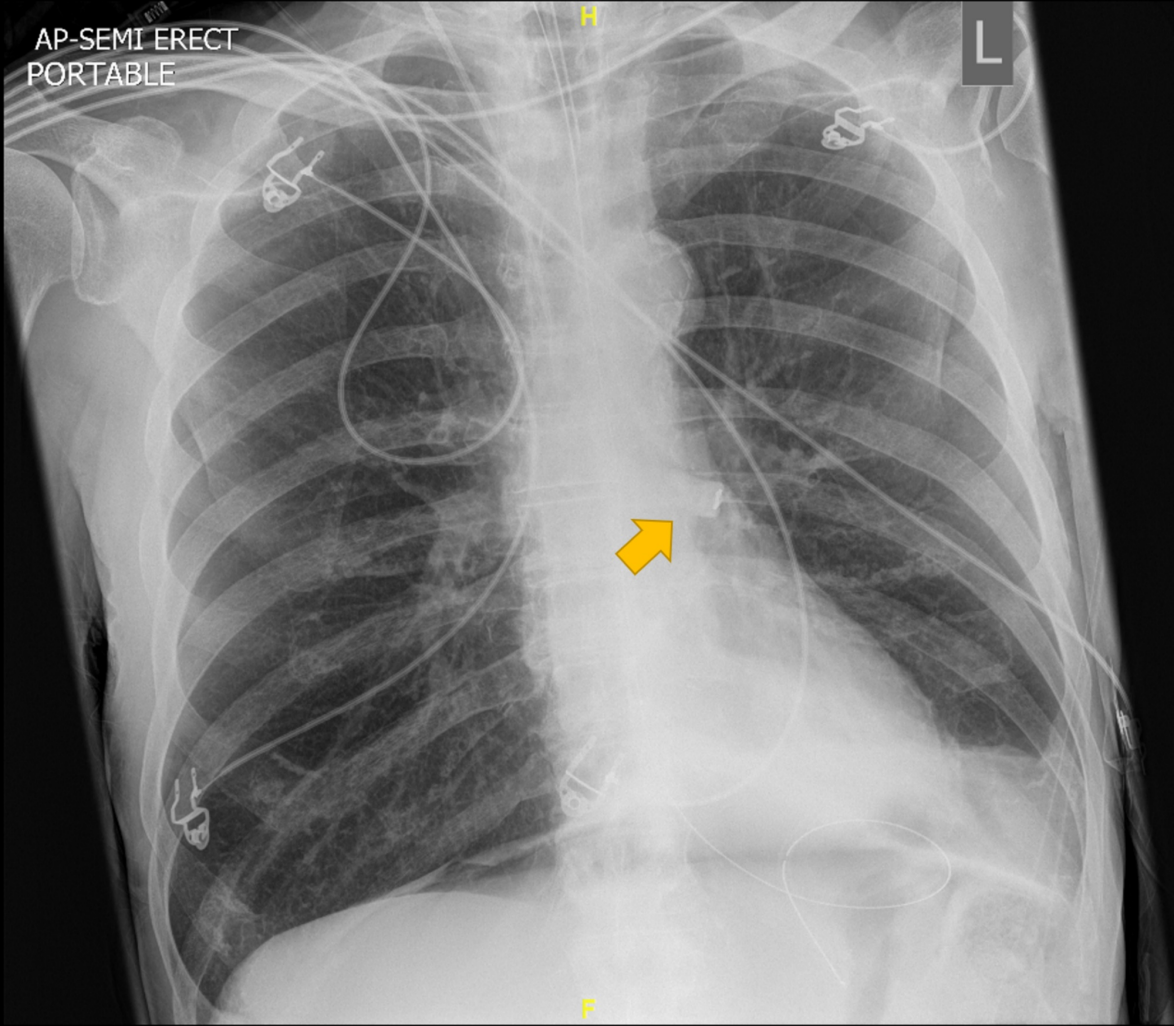
Radiograph showing a tooth in the left main bronchus.

A chest X-ray, shown in Figure 2, at post-intubation showed what was, in retrospect, a molar with a ceramic filling in the left main bronchus; however, this was not recognised at the time.

Advice was sought from a respiratory consultant who performed flexible bronchoscopy that afternoon. His findings were documented as ‘left main bronchus partially occluded by poorly vascular polypoid necrotic lesions fixed to bronchus’. Washings were sent and biopsies were performed.

**Figure 3:**
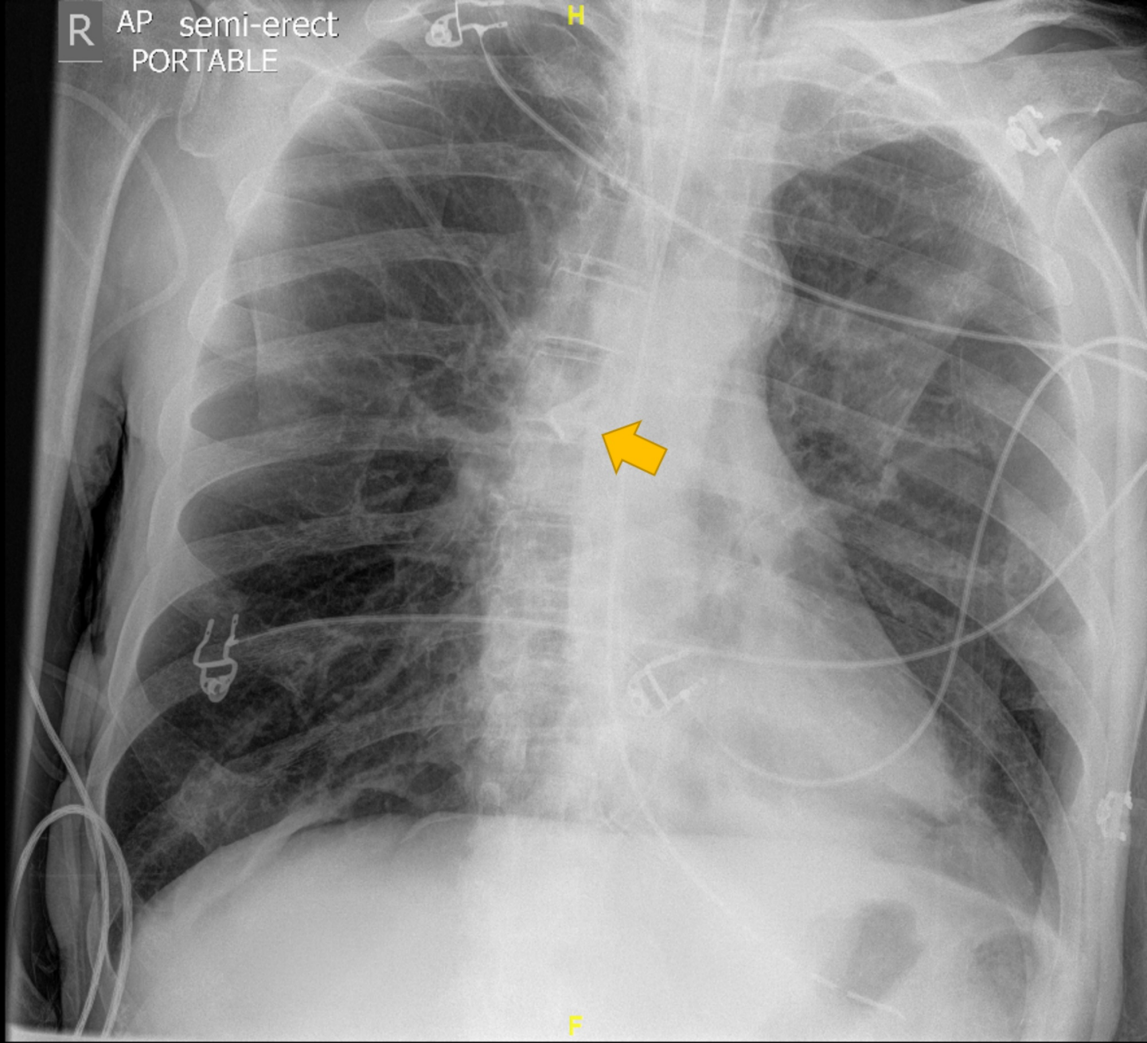
Plain radiograph demonstrating a tooth in the right main bronchus.

A repeat chest X-ray that evening at 23:09 showed the tooth had moved from the left main bronchus to the right, as seen in Figure 3. Again, the tooth was not identified. The next morning, the patient was extubated. The tooth was found incidentally at the end of the endotracheal tube’s suction catheter. A post-extubation film demonstrated no tooth in situ. A subsequent review of the two previous X-rays revealed the tooth.

The patient recovered well, with no further complications, and was later discharged home.

## Discussion

Dental injury is one of the well-recognised complications of tracheal intubation. This is due to several causative factors, including the anterior teeth being used unintentionally for support and biting on the endotracheal tube. The frequency of dental injury has been shown to be between 0.17% and 12.1%, with an increase in frequency where there is limited mouth opening, limited mobility of the mandibular area and neck, poor visibility of the hypopharynx, and small thyromental distance. Additionally, poor dental health can further increase the risk of tooth in lung events. These include the presence of dental caries, marginal periodontitis, insufficient restorations, and existing ceramic restorations [1].

Tooth aspiration can cause airway obstruction and asphyxiation, atelectasis, inability to clear secretions, and secondary infection [2]. Factors that can increase the risk of aspiration of foreign bodies include facial trauma, seizures, intoxication, dental procedures, and altered consciousness [3]. In cases of severe trauma, an aspirated foreign body may be undiagnosed for weeks to months [4]. The diagnosis of a foreign body in semi-conscious or unconscious patients can be difficult, as they may be asymptomatic. However, the symptoms of an aspirated foreign body can include choking, haemoptysis, wheezing, coughing, and shortness of breath. In ventilated patients, the signs may be more subtle, including monophonic wheeze, reduced breath sounds, or sudden increase in FiO2 requirement or ventilation pressures [3].

Chest radiographs are useful in identifying tooth aspiration, however, teeth overlying the mediastinum or cardiac muscle may be interpreted as an artefact or not seen at all. On occasion, chest computed tomography (CT) is required to make the diagnosis [5].

In this instance, endoscopic retrieval was not necessary. In cases where it is, flexible bronchoscopy is becoming increasingly successful. However, rigid bronchoscopy may still be required. Both approaches use an oral as opposed to a nasal approach. Foreign bodies that lie in the distal airways are more suited to a flexible bronchoscope. Generally, grasping forceps or rubber tip forceps are used to retrieve hard foreign bodies. However, given the wide, round shape of a tooth, a basket or snare retrieval device may provide better purchase [6].

The location, surface and shape of the foreign body influence how successful these procedures can be. In some cases, it may be necessary to undertake an open thoracotomy, however, it is noted that this should only be used in extremis [7].

In this case, the tooth was able to be removed using deep suction and removal of the endotracheal tube. It highlights how a detailed interpretation of chest radiographs by experienced practitioners should be used to identify foreign bodies early in the clinical course and prevent misdiagnosis.

## Conclusions

An elective surgical patient with poor dentition was intubated three times. The morning after the second extubation, he desaturated likely due to the aspiration of a tooth and build-up of secretions behind it. Initially thought to be a neoplasm on bronchoscopy, repeat chest X-rays clearly show an aspirated tooth; however, this was not recognised at the time. The tooth was extracted unintentionally with suction during his third and final extubation. The patient made a full recovery.
